# Small non-coding RNA *fen36*: a novel positive regulator of biofilm formation and swarming motility in *Bacillus amyloliquefaciens*

**DOI:** 10.1128/spectrum.04194-25

**Published:** 2026-05-29

**Authors:** Hedong Lu, Chengxin Jiang, Yi Liu, Panping Yang, Yan Liu, Jiao Ge, ZhaoChuan Dong, Yuping Zhao, Chengxin Geng

**Affiliations:** 1 School of Life Science and Food Engineering, Huaiyin Institute of Technology 66520, Huaian, China; 2 Jiangsu Vocational College of Agriculture and Pastoral Industry, Taizhou, China; Reichman University, Herzeliya, Israel

**Keywords:** biofilm, sRNA, RNA-seq, *Bacillus amyloliquefaciens*

## Abstract

Biofilm formation is a critical developmental process for the survival of *Bacillus*, its environmental adaptation, and host colonization. This study investigated the regulatory function of the novel small non-coding RNA (sRNA) *fen36* in biofilm architecture and swarming motility in *Bacillus amyloliquefaciens*. A comparative transcriptomic analysis was carried out between the wild-type *B. amyloliquefaciens* LPB-18 and a *fenSr3* deletion mutant (LPB-18N). This analysis, integrated with IntaRNA-based thermodynamic predictions, identified *fen36* as a highly upregulated sRNA. Subsequently, functional assays utilizing isogenic *fen36*-knockout (LPB-18NΔ*fen36*) and overexpression (LPB-18N::*fen36*) strains established *fen36* as a positive regulator of biofilm formation. Specifically, the overexpression of *fen36* yielded a 3.59-fold increase in biofilm biomass, induced a hyper-swarming phenotype, and generated highly wrinkled biofilm topologies, as confirmed by scanning electron microscopy (SEM). Crucially, these phenotypic enhancements occurred without compromising planktonic growth kinetics or the biosynthesis of the antifungal lipopeptide fengycin. *In vitro* dual-culture assays further demonstrated that the *fen36* overexpression strain maintained potent antagonistic efficacy against the phytopathogen *Fusarium oxysporum*. Mechanistic investigations employing a dual-plasmid reporter system and RT-qPCR revealed that *fen36* targets the 5′-untranslated region of *tasA*, upregulating this core matrix gene. Furthermore, the transcription of *fen36* is negatively regulated by the stress-responsive sRNA *fenSr3*. Collectively, these findings elucidate a novel *fenSr3-fen36-tasA* regulatory cascade that governs biofilm architecture and motility independent of secondary metabolism, offering a precise genetic target for optimizing Bacillus biocontrol performance.

**IMPORTANCE:**

*Bacillus amyloliquefaciens* is extensively harnessed in agriculture for its robust rhizosphere colonization and antimicrobial lipopeptide synthesis. Understanding the genetic networks uncoupling physical colonization from secondary metabolism is critical for engineering superior biocontrol agents. This study elucidates a novel post-transcriptional regulatory cascade, fenSr3-fen36-tasA, governing multicellular behavior. The newly identified sRNA fen36 significantly enhances biofilm formation and hyper-swarming motility by upregulating the matrix gene tasA. Crucially, this enhancement occurs without disrupting fengycin biosynthesis, maintaining potent antagonism against phytopathogens such as *Fusarium oxysporum*. By mapping this dual-sRNA hierarchy, our research provides crucial mechanistic insights into bacterial environmental adaptation, offering refined genetic targets to optimize *Bacillus* strains for sustainable agricultural applications.

## INTRODUCTION

*Bacillus amyloliquefaciens* has emerged as a versatile chassis cell and a prominent model organism in contemporary microbiological and bioengineering research (1). As a Gram-positive, facultative anaerobe, and spore-forming bacterium belonging to the genus *Bacillus*, it exhibits robust capabilities for both sporulation and biofilm development (2). This rod-shaped bacterium is ubiquitous across diverse ecological niches, successfully colonizing terrestrial habitats, aquatic systems, plant phyllospheres, animal gastrointestinal tracts, and various food matrices. Notably, *B. amyloliquefaciens* exhibits exceptional resilience to environmental stress, conferring a significant competitive advantage under adverse conditions (3). Confirming its non-pathogenicity based on its “Generally Recognized as Safe” (GRAS) status, this microbe is extensively harnessed in food biotechnology, pharmaceutical manufacturing, agricultural biocontrol, and environmental bioremediation (4).

*B. amyloliquefaciens* serves as a valuable probiotic starter culture and additive in industrial fermentation, producing an array of digestive enzymes that facilitate the efficient breakdown of complex carbohydrates and proteins (5). However, diminished metabolic activity and high mortality rates under fermentation stress have long impeded the broader industrial applications of these functional strains (6). Multicellular biofilm formation plays a critical role in mitigating these challenges. The biofilm matrix not only confers structural stability but also shields the encased bacterial cells from common fermentation-induced adversities, such as oxidative stress and nutrient depletion (7). Furthermore, robust biofilm development facilitates persistent adhesion to solid substrates, a prerequisite for long-term cell viability and continuous bioprocessing (8). Therefore, understanding the metabolic mechanisms underlying biofilm formation and optimizing its synthesis pathway are essential steps to enhance the survival and productivity of fermentation strains (9). By modulating biofilm synthesis, microbial stress tolerance can be improved, fermentation characteristics can be optimized, and the longevity of strains can be extended (10). Among the diverse secondary metabolites secreted by *B. amyloliquefaciens*, the antifungal compound fengycin has garnered significant attention. Fengycin is a cyclic lipopeptide synthesized via a non-ribosomal peptide synthetase (NRPS) mechanism encoded by the *fen* operon (11). Beyond its potent antagonistic activity against filamentous fungi, fengycin contributes to biofilm structural integrity by modulating cell surface hydrophobicity and intercellular adhesion (11). Recent studies demonstrated that metabolic engineering or the modification of biofilm-associated genes can significantly enhance fengycin yield in *B. amyloliquefaciens*, underscoring an indicative functional link between secondary metabolite biosynthesis, multicellular behavior, and environmental adaptability (12, 13). In agricultural contexts, *B. amyloliquefaciens* serves as a highly effective plant growth-promoting agent. Beyond secreting phytohormones, amino acids, and extracellular enzymes, the bacterium establishes mutually beneficial interactions with plant roots via robust biofilm-mediated rhizosphere colonization. This physical interaction not only facilitates nutrient acquisition but also provides a biocontrol shield against soil-borne phytopathogens (14). Ultimately, these synergistic benefits enhance crop yield and stress resilience, enhanced stress resistance, and a reduction in the use of fertilizers and pesticides, ultimately supporting the development of sustainable agriculture (15).

Small non-coding RNAs (sRNAs) play pivotal roles in orchestrating diverse metabolic processes, encompassing biofilm formation, stress response, and bacterial adaptation to environmental fluctuations (16). Recent studies have shown that sRNAs help bacteria fine-tune gene expression in response to environmental signals, such as nutrient availability and cell density, which directly impact biofilm formation and matrix synthesis (17). In *B. amyloliquefaciens*, the sRNA *PhoS* is induced during phosphate starvation and promotes biofilm formation by upregulating the expression of matrix operons, including *epsA-O* and *tapA-sipW-tasA* via the transcriptional regulator PhoP (18, 19). Furthermore, sRNAs frequently interact with master regulatory circuits, such as Spo0A and CodY, to modulate multicellular behavior in response to fluctuating environmental conditions. This interaction forms a complex regulatory network that controls the biofilm lifecycle, from initiation to maturation (20). Parallel to these structural components, the lipopeptide surfactin serves as a critical signaling molecule for biofilm initiation in *Bacillus* species, and its biosynthesis is similarly subject to sRNAs-mediated regulation. Specifically, the production of surfactin is tightly controlled by sRNAs that modulate the transcription of the *srfA* operon (21). Collectively, these studies highlight the dynamic and multifaceted role of sRNAs in regulating biofilm formation, underscoring their potential as targets for biofilm-based applications in biotechnology and agriculture.

In our previous work, we identified the sRNA *fenSr3* via comparative transcriptomic analysis, demonstrating that a ∆*fenSr3* knockout strain exhibited markedly enhanced biofilm formation and fengycin biosynthesis. These findings underscored *fenSr3* as a key post-transcriptional regulator influencing both secondary metabolism and environmental adaptability in *B. amyloliquefaciens* (22). *Bacillus species* integrate the regulation of biofilm formation and swarming motility through interconnected transcriptional networks, primarily governed by the master regulators Spo0A, SinR/SinI, and DegU (23, 24). These regulatory hubs form a bistable decision-making switch, enabling the bacterial population to transition seamlessly between sessile surface colonization and motile dispersal. Building upon this framework, we hypothesized that the newly identified sRNA *fen36* functions as a critical regulatory node within a *fenSr3*-dependent sRNA hierarchy, modulating both matrix gene transcription and swarming behavior. Therefore, the present study investigated the regulatory mechanisms of sRNA *fen36* in *B. amyloliquefaciens* LPB-18N biofilm formation. This comprehensive analysis was achieved by integrating comparative transcriptome sequencing, bioinformatics predictions, and targeted molecular functional validation, and was further supported by physiological and biochemical analyses.

## RESULTS

### Comparative analysis of biofilm formation capacity among derivative strains

**Fig 1:**
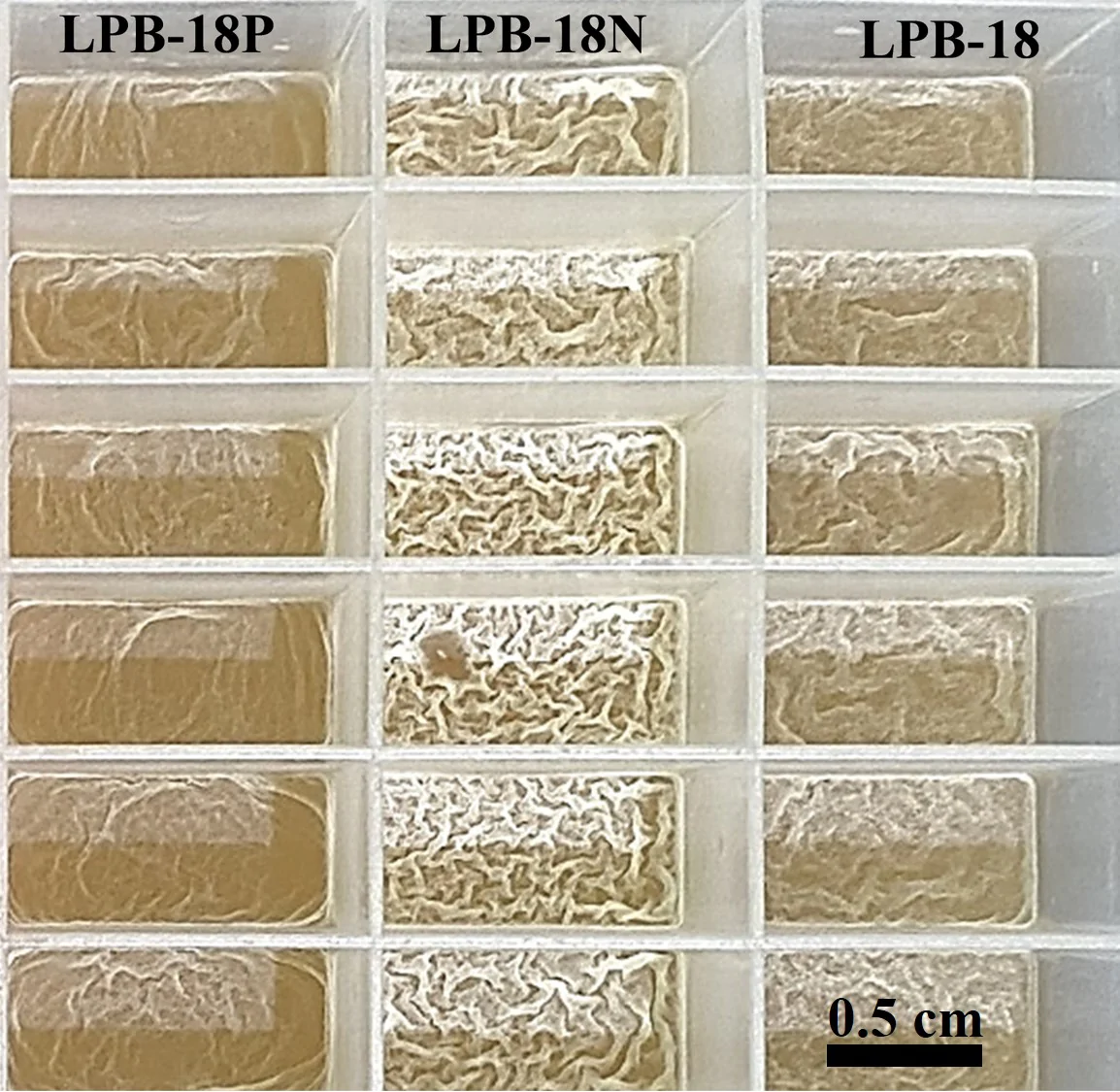
Top-view of 48-h bacterial biofilms from different strains cultured statically. The figure shows a top-down view of biofilms for the three strains at 48 h of static cultivation, with LPB-18P having the weakest biofilm formation ability and LPB-18N the strongest. Scale bar = 0.5 cm. Each column represents six independent biological replicates performed under identical conditions. The observed biofilm morphotypes were consistent across all replicates.

To compare the biofilm-forming capacities of the wild-type LPB-18 and its derivative strains LPB-18N and LPB-18P, macroscopic top-view images of biofilms cultured in 96-well plates were captured (Fig. 1). The assay revealed distinct morphological differences across the tested strains. Strain LPB-18P produced a fragile and relatively unstructured biofilm, whereas the LPB-18N mutant developed a robust, highly wrinkled topography with a well-defined three-dimensional architecture. In contrast to LPB-18N, both the wild-type LPB-18 and LPB-18P strains exhibited reduced biofilm biomass during the mid-to-late stages of static culture. Collectively, these observations demonstrate that the genetic modifications in the derivative strains profoundly alter biofilm morphotypes and developmental dynamics.

### Isolation and characterization of sRNA *fen36*

#### Enrichment analysis of differentially expressed sRNAs

**Fig 2:**
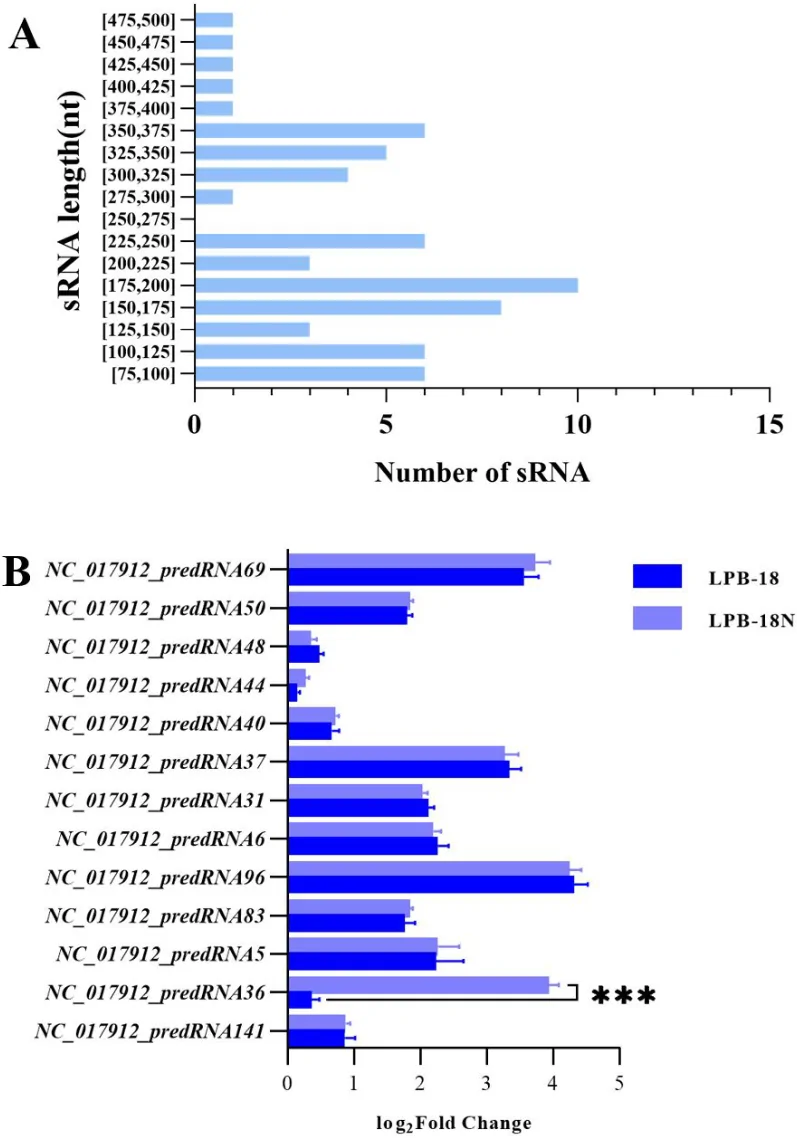
Identification and relative expression of small non-coding RNAs (sRNAs) associated with biofilm formation in *B. amyloliquefaciens*. (**A**) Genome-wide prediction of intergenic small RNAs using the Rockhopper pipeline. Predicted sRNAs are plotted according to chromosomal location and transcript length (bp). The inset shows an expanded view of the genomic region containing *fen36*. (**B**) Relative expression levels of selected sRNAs in LPB-18 (wild type), LPB-18N (Δ*fenSr3* mutant), and LPB-18P (*fenSr3* overexpression strain) as determined by RT-qPCR. Expression values were normalized to 16S rRNA and are presented as mean ± SD from six independent biological replicates (*n* = 3). Each bar represents the average of three replicates, and individual data points are shown. Statistical analysis was performed using one-way ANOVA with Tukey’s post hoc test; different letters above the bars indicate significant differences (***P < 0.001).

Transcripts from the newly identified intergenic regions, as determined by Rockhopper, were annotated against the non-redundant (nr) database and subsequently filtered to exclude sequences shorter than 50 nucleotides (nt). In total, 68 candidate sRNAs were detected, and their length distribution is presented in Fig. 2A. The identified sRNAs ranged from 75 to 500 nucleotides, with the majority concentrated between 175 and 200 nt. Among them, the largest group contained 10 predicted sRNAs. To further refine these candidates, IntaRNA 2.0.3, combined with Pearson’s correlation coefficient, was employed to predict differentially expressed sRNAs, followed by comprehensive target analysis.

#### Bioinformatics analysis of sRNAs related to the regulation of biofilm synthesis

Analysis using IntaRNA 2.0.3 to predict potential molecular targets of sRNA *fenSr3* suggested a low likelihood of direct regulation of biofilm synthesis. This result implied that *fenSr3* may influence biofilm formation indirectly by modulating other genes. To further assess the binding potential of sRNAs to biofilm-related genes, we performed enrichment analysis on the full set of 68 candidate sRNAs identified earlier. IntaRNA was utilized to predict thermodynamic interactions between these candidate sRNAs and core genes governing biofilm matrix production, specifically *tasA*, *tapA*, *blsA*, and *eps* operon. Given that sRNAs predominantly base-pair with the 5′-untranslated regions (5′ UTRs) of their target mRNAs, our computational screening was strictly focused on the translational initiation regions (spanning nucleotides −39 to +19 relative to the start codon). This analysis identified five candidate sRNAs potentially associated with biofilm synthesis: NC_017912_predRNA141, NC_017912_predRNA36, NC_017912_predRNA5, NC_017912_predRNA83, and NC_017912_predRNA9 (Table 1).

**TABLE 1:** Predicted results of IntaRNA 2.0.3 program

sRNA_id	mRNA_id	Energy (kcal/mol)	sRNA_ position	mRNA_ position	Symbol
NC_017912_predRNA141	MUS_RS12880	−6.90945	108.135 (27)	11.43 (32)	sipW
NC_017912_predRNA36	MUS_RS12875	−17.3627	68.173 (105)	4.113 (109)	*tasA*
NC_017912_predRNA5	MUS_RS12875	−7.59749	61.68 (7)	3.11 (8)	*tasA*
NC_017912_predRNA83	MUS_RS12880	−4.9486	52.68 (16)	16.36 (20)	sipW
NC_017912_predRNA96	MUS_RS12875	−14.6822	22.46	1.29	*tasA*

To ensure the reliability of these predictions, Pearson correlation coefficients were calculated to evaluate the relationship between the expression levels of candidate sRNAs and their corresponding genes across all samples, with *P*-values used to assess statistical significance (Table 2). Applying a strict screening threshold (*P* < 0.05) to denote significant correlations. This approach ultimately identified 14 sRNAs linked to biofilm formation. To validate these predictions, we compared the expression profiles of the candidate sRNAs in the wild-type strain LPB-18 and the *fenSr3*-deficient strain LPB-18N (Fig. 2B). Among them, NC_017912_predRNA36 (hereafter designated *fen36*) showed markedly different transcript levels between the two strains. Correlating this robust transcriptional upregulation with the pronounced biofilm enhancement observed in the mutant, we hypothesized that *fen36* functions as a critical positive regulator of biofilm matrix biosynthesis.

**TABLE 2:** Pearson correlation coefficient calculation prediction

Gene	sRNA	Cor	*P* value
MUS_RS08900 (hfq)	NC_017912_predRNA6	0.051475	0.722856
MUS_RS12880(sipW)	NC_017912_predRNA31	−0.36319	0.479168
MUS_RS12875(*tasA*)	NC_017912_predRNA36	−0.997574	0.003215
MUS_RS12880(sipW)	NC_017912_predRNA36	−0.982182	0.000517
MUS_RS12875(*tasA*)	NC_017912_predRNA37	−0.87074	0.023982
MUS_RS12880(sipW)	NC_017912_predRNA40	−0.88112	0.020357
MUS_RS12875(blsA)	NC_017912_predRNA40	−0.83105	0.040406
MUS_RS12880(sipW)	NC_017912_predRNA44	−0.83019	0.040805
MUS_RS12875(*tasA*)	NC_017912_predRNA46	−0.8556	0.029774
MUS_RS17435(comP)	NC_017912_predRNA48	−0.85744	0.029038
MUS_RS12980(spo0A)	NC_017912_predRNA50	−0.26006	0.618705
MUS_RS17455(epsJ)	NC_017912_predRNA53	−0.22203	0.672423
MUS_RS17480(epsC)	NC_017912_predRNA69	0.35855	0.999214

### Functional validation of sRNA *fen36* and phenotypic analysis of mutant strains

#### Measurement of biofilm formation capacity

**Fig 3:**
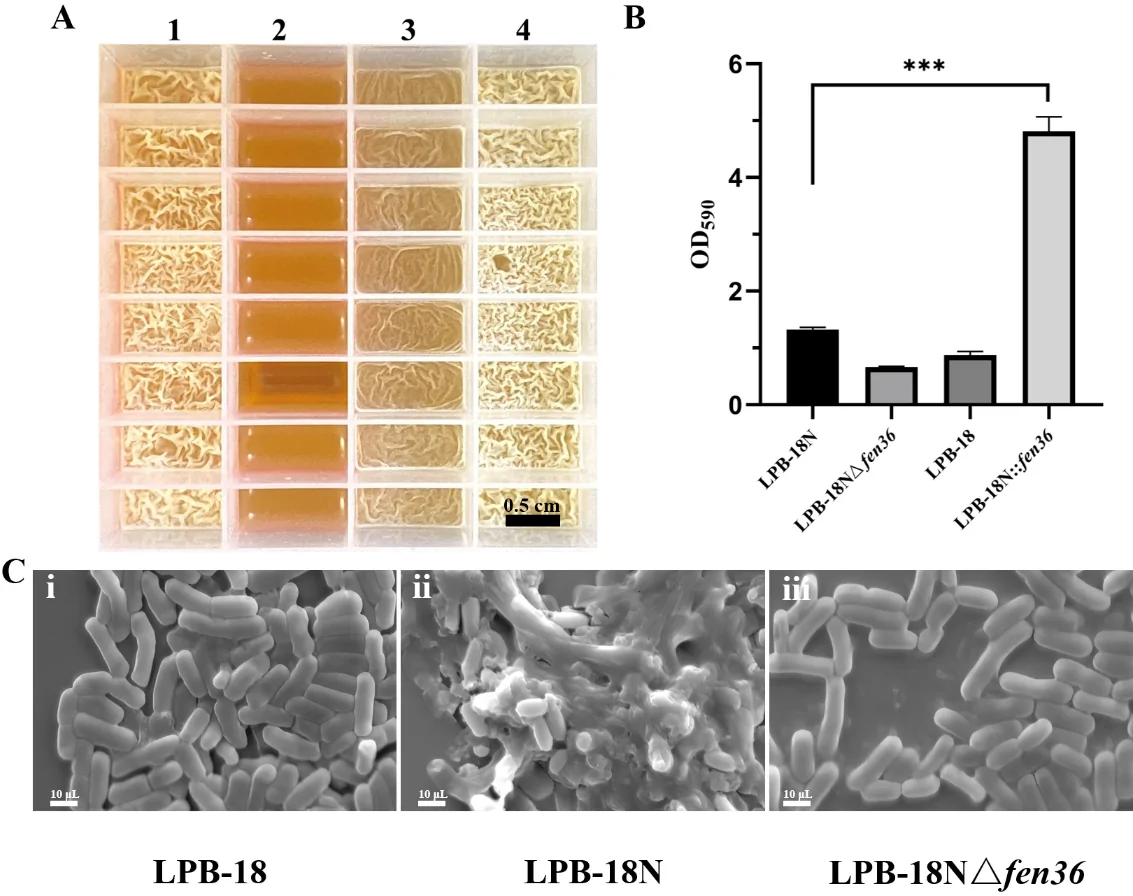
Phenotypic characterization of *fen36* knockout and overexpression strains in *B. amyloliquefaciens*. (**A**) Top-view morphology of 48-h static biofilms formed by different strains in 96-well plates. Strains are as follows: (1) LPB-18 (wild-type), (2) LPB-18N (*fenSr3* knockout), (3) LPB-18N∆*fen36* (*fenSr3, fen36* double knockout), and (4) LPB-18N::*fen36* (*fenSr3* knockout with IPTG-induced *fen36* overexpression). Images are representative of eight independent biological replicates (Scale bars = 0.5 cm). (**B**) Semi-quantitative analysis of biofilm biomass measured by crystal violet staining and absorbance at 590 nm. Data are presented as mean ± SD of three biological replicates (*n* = 3). Individual data points are shown. Statistical significance was determined by one-way ANOVA with Tukey’s post-hoc test (***P < 0.001). (**C**) Scanning electron micrographs (SEM) of biofilm surface structures after 36 h of growth. Panels show representative images of: (i) LPB-18; (ii) LPB-18N, and (iii) LPB-18N∆*fen36* (Scale bars = 10 µm).

Four strains were utilized for a comparative analysis of biofilm formation: the wild-type LPB-18, the *fenSr3* deletion mutant LPB-18N, the Δ*fen36* Δ*fenSr3* double mutant LPB-18NΔ*fen36*, and LPB-18N::*fen36*, a *fenSr3*-deficient strain carrying an inducible *fen36* expression construct. The sRNA (*fen36*) knockout and overexpression strains LPB-18N∆*fen36* and LPB-18N::*fen36* were constructed using homologous recombination techniques. As shown in Fig. 3A, these strains exhibited distinct differences in biofilm formation. Strain LPB-18N developed biofilms with pronounced folds in 96-well plates, whereas the wild-type strain produced loose biofilms with surfaces that were easily detached, consistent with previous observations. By contrast, the LPB-18NΔ*fen36* mutant merely generated flocculent biofilm fragments that failed to aggregate into a cohesive interfacial membrane. Conversely, the overexpression strain LPB-18N::*fen36*, induced by IPTG, formed dense, compact, three-dimensional biofilms. Semi-quantitative measurements of biofilm production across the strains (Fig. 3B) revealed that *fen36* overexpression increased biofilm synthesis by 3.59-fold under IPTG induction, while *fen36*-deficient strains displayed significantly reduced biofilm-forming capacity, reverting to levels comparable to the wild-type strain. These findings strongly suggested that sRNA *fen36* serves as a key positive regulator of biofilm synthesis in *B. amyloliquefaciens* LPB-18N.

#### Electron microscopy of biofilm formation

To examine the microstructural differences among strains, scanning electron microscopy (SEM) was used to analyze the cell surface morphology of *B. amyloliquefaciens* LPB-18 and its mutant derivatives (LPB-18N and LPB-18N∆*fen36*). As shown in Fig. 3C through E, biofilms observed after 36 h display clear strain-specific variations. Strain LPB-18N formed a thin surface film with distinct ductile folds and a complex three-dimensional architecture. In contrast, the wild-type LPB-18 primarily exhibited a smoother surface with less prominent folds. Strikingly, SEM micrographs of the *fen36* knockout strain (LPB-18NΔ*fen36*) revealed cells with a typical rod-shaped morphology, bluntly rounded poles, and smooth surfaces, yet lacking robust matrix interconnectivity. A thin surface layer was observed within the surrounding intercellular space, consistent with partial detachment of surface-associated material. These results indicated that biofilm formation capacity varied among the strains, with LPB-18N showing the highest capacity, followed by LPB-18 and LPB-18N∆*fen36* exhibiting the lowest capacity. Notably, while deletion of sRNA *fenSr3* enhanced biofilm biosynthesis, the opposite effect was observed in *fen36*-deficient strains. Together, these findings suggested that sRNA *fen36* serves as a key regulatory factor essential for biofilm biosynthesis in *B. amyloliquefaciens* LPB-18N, positively controlling its formation.

### Phenotypic characterization

#### Growth curve measurement

**Fig 4:**
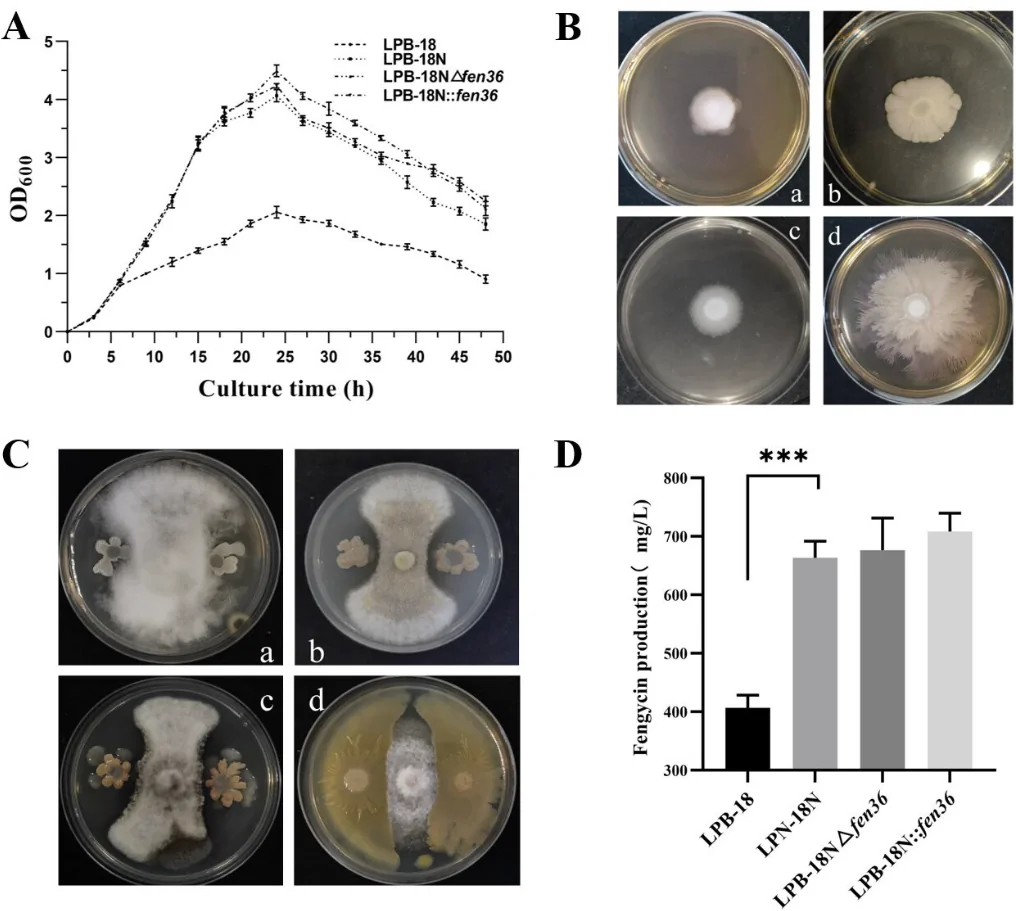
Analysis of physiological phenotypes.(**A**) Growth curves of different mutant strains. (**B**) The mobility test of different strains. (a) LPB-18 (*d* = 0.98 cm); (b) LPB-18N (*d* = 2.23 cm); (c) LPB-18N∆*fen36* (*d* = 1.11 cm); (d) LPB-18N::*fen36* (*d* = 5.41 cm). (**C**) The inhibitory capacity analysis of different strains against *Fusarium oxysporum*. a to d represent LPB-18, LPB-18N, LPB-18N∆*fen36,* and LPB-18N::*fen36*, respectively. (**D**) Fengycin yield in different strains (*P* < 0.001). Statistical significance was determined by one-way ANOVA with Tukey’s post hoc test (***P < 0.001). Strain abbreviations: LPB-18, wild-type *B. amyloliquefaciens*; LPB-18N, *fenSr3* deletion mutant; LPB-18NΔ*fen36*, *fen36* deletion in the LPB-18N background; LPB-18N::*fen36*, *fenSr3*-deficient strain carrying an inducible *fen36* expression construct.

To ascertain whether the observed variations in biofilm formation were confounded by inherent strain-specific growth defects, the planktonic biomass accumulation of the various *B. amyloliquefaciens* strains was monitored over a 48-h period (Fig. 4A). Comparison between the wild-type strain LPB-18 and the derivative strain LPB-18N revealed higher biomass accumulation in LPB-18N. In contrast, the *fen36* knockout (LPB-18N∆*fen36*) and overexpression (LPB-18N::*fen36*) strains exhibited no significant differences in biomass compared with LPB-18N. These results indicated that *fen36* does not exert a substantial regulatory effect on the overall growth of *B. amyloliquefaciens* LPB-18N.

#### Effect of the sRNA *fen36* on swarming motility

Bacteria employ flagellar motility to colonize ecological niches, enabling them to evade environmental stresses and adapt to changing conditions. In this study, wild-type and *fen36*-variant strains were inoculated into semi-solid LB medium, and their movement patterns were recorded after 24 h (Fig. 4B). At the solid–air interface, strains LPB-18 and LPB-18N exhibited limited motility, whereas the overexpression strain LPB-18N::*fen36* showed strong surface attachment and pronounced motility. Specifically, LPB-18N::*fen36* exhibited a distinct radial spreading trajectory, rapidly colonizing the semi-solid surface to form a motility halo with a diameter of 5.42 cm. In contrast, the *fen36* knockout strain LPB-18N∆*fen36* exhibited markedly reduced motility, with a motility circle diameter of only 1.11 cm.

#### Testing of sRNA *fen36* for antagonistic ability against pathogenic bacteria

To explore the agricultural potential of these strains, the phytopathogenic fungus *Fusarium oxysporum* was utilized as an indicator organism to evaluate biocontrol efficacy via *in vitro* dual-culture plate confrontation assays (Fig. 4C). The wild-type strain LPB-18 exhibited only weak antagonistic activity against *Fusarium oxysporum*, producing an inhibition zone of 2 cm in diameter. In contrast, the three mutant strains (LPB-18N, LPB-18N::*fen36*, and LPB-18N∆*fen36*) showed strong inhibitory effects under the same conditions, with an average inhibition zone diameter of 5.04 ± 0.37 cm. Differences among the mutant strains were not statistically significant. Notably, during co-culture with *Fusarium oxysporum*, the LPB-18N::*fen36* strain demonstrated potent biofilm-forming ability, which allowed it to effectively occupy the ecological niche of the pathogen, thereby displaying an antagonistic effect comparable to that of the other mutant strains. To further ascertain whether the sRNA *fen36* pleiotropically influences secondary metabolism, fengycin production was quantitatively analyzed across the tested strains (Fig. 4D). The results indicated no statistically significant differences in yield among the mutants.

### The mechanism of sRNA *fen36*-mediated biofilm formation

#### Analysis of molecular targets of sRNA

**Fig 5:**
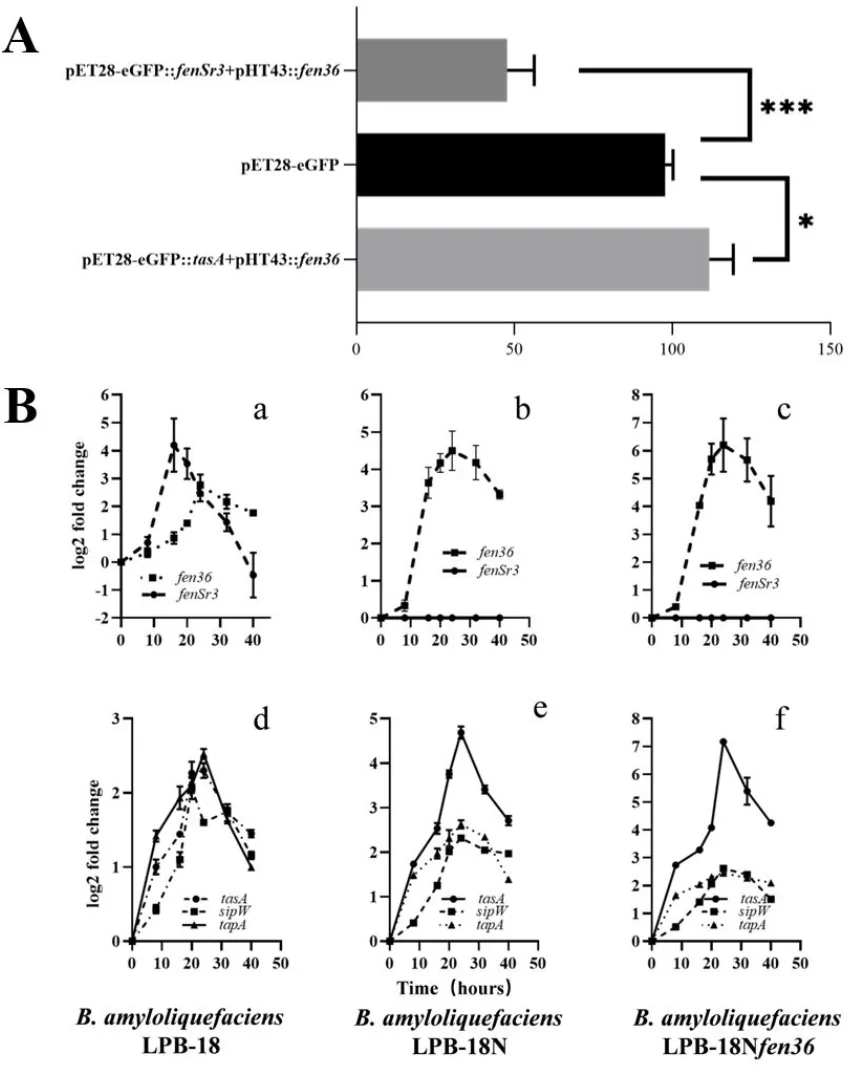
Mechanistic analysis of sRNA *fen36* in regulating biofilm formation. (**A**) sRNA target verification results (pET-28-eGFP was blank control). Data are presented as relative fluorescence units (RFU) normalized to optical density at 50 nm (RFU/OD_540_). Each bar represents the mean ± SD from six independent biological replicates (*n* = 3). Statistical significance was determined using one-way ANOVA followed by Tukey’s multiple-comparison test (*P < 0.05 and ***P < 0.001). (**B**) Expression levels of sRNA and target genes detected by RT-qPCR. a and d represent LPB-18; b and e represent LPB-18N; c and f represent LPB-18N::*fen36*. Strain abbreviations: LPB-18, wild-type *B. amyloliquefaciens*; LPB-18N, *fenSr3* deletion mutant; LPB-18NΔ*fen36*, *fen36* deletion in the LPB-18N background; LPB-18N::*fen36*, *fenSr3*-deficient strain carrying an inducible *fen36* expression construct.

The dual-plasmid systems pET28-eGFP::*tasA*/pHT43::*fen36* and pET28-eGFP::*fenSr3*/pHT43::*fen36* were induced by IPTG and cultured overnight. Fluorescence intensity at 540 nm was then measured using a multi-mode microplate reader, and the results are shown in Fig. 5A. In the pET28-eGFP::*tasA*/pHT43::*fen36* system, the enhanced fluorescence signal suggested a potential regulatory interaction between *fen36* and *tasA*. Together with the phenotypic analysis of LPB-18N::*fen36*, these results suggest that *fen36* may be involved in the regulation of *tasA* transcription and thereby enhance biofilm synthesis. In contrast, the pET28-eGFP::*fenSr3*/pHT43::*fen36* system exhibited reduced fluorescence, implying mutual repression between sRNA *fenSr3* and *fen36*. Considering *fen36*’s biofilm-promoting role (with LPB-18N::*fen36* showing a 3.59-fold increase in biofilm formation) and the strong biofilm-forming capacity of the *fenSr3*-deficient strain LPB-18N, we proposed that *fenSr3* inhibits *fen36* transcription. This regulatory relationship was preliminarily confirmed using the dual-fluorescence system and is consistent with the biofilm phenotypes observed across the different strains.

#### Mechanistic analysis of sRNA *fen36* in regulating biofilm formation

To further investigate the regulatory role of sRNA *fen36* in controlling the amyloid fiber protein-coding gene *tasA* and its interaction with other sRNAs, the transcript levels of *fen36*, *fenSr3*, and the *tasA-sipW-tapA* cluster were monitored at 8-h intervals in strains LPB-18, LPB-18N, and LPB-18N::*fen36* (Fig. 5B). RT-qPCR analyses were performed using 16S rRNA as the internal reference gene, and all measurements represent the mean ± SD from three independent biological replicates (*n* = 3). Statistical analyses were performed using one-way analysis of variance (ANOVA) followed by Tukey’s post hoc test. All quantitative data are presented as mean ± standard deviation (SD). Differences were considered statistically significant at *P* < 0.05. In the wild-type strain LPB-18, *fenSr3* transcripts were abundant during the exponential phase but gradually declined as cells entered the stationary phase. In contrast, *fen36* transcripts were relatively low during the exponential phase but remained stable during the stationary phase. To determine whether *fenSr3* regulates *fen36* expression, *fen36* transcript levels were quantified in the *fenSr3* knockout strain LPB-18N. A significant increase was observed, indicating that *fenSr3* suppresses *fen36* expression or reduces its transcript stability, thereby lowering its abundance. Transcript levels of the *tasA-sipW-tapA* operon also varied among strains. In wild-type LPB-18, *tapA* expression remained low, possibly due to negative regulation by *fenSr3*. In the *fenSr3* knockout strain LPB-18N, *tasA* transcription increased twofold, consistent with phenotypic assays showing enhanced biofilm formation relative to the wild type. Computational predictions suggested only weak binding potential between *fenSr3* and *tasA*, indicating that *fenSr3* likely regulates biofilm formation indirectly. In the overexpression strain LPB-18N::*fen36*, *tasA* expression was markedly upregulated, whereas *sipW* and *tapA* showed no significant changes. Together, data from the dual-plasmid system and RT-qPCR analyses suggested that *fen36* may directly bind to *tasA*, thereby promoting its transcription and enhancing biofilm synthesis.

## DISCUSSION

Biofilm formation is fundamental to the biological functions of *Bacillus amyloliquefaciens*, particularly in environmental adaptation, host colonization, and ecological interactions (25). By secreting an extracellular polymeric substances (EPS) matrix, biofilms provide architectural stability and facilitate cooperative behaviors that enhance bacterial survival under stress conditions, such as oxidative stress, nutrient limitation, and desiccation (26). In agriculture, biofilms formed by *B. amyloliquefaciens* support root colonization, suppress phytopathogens, and facilitate nutrient solubilization, thereby improving plant growth and soil health (27). Beyond agriculture, biofilms also contribute to bioremediation by adsorbing environmental pollutants, including dyes and heavy metals, through electrostatic interactions and functional groups (e.g., carboxyl, amine, and phosphate) present in EPS (28). In this study, we demonstrated that *fen36* enhances both biofilm synthesis and bacterial motility without affecting fengycin production, underscoring its potential as a regulatory target for optimizing beneficial traits in biotechnological applications.

Small RNAs (sRNAs), typically 50–500 nucleotides in length, play pivotal roles in bacterial metabolism by regulating key processes, such as cell division, quorum sensing, stress responses, and virulence (29). These regulatory molecules exert their effects primarily through interactions with target mRNAs or proteins, thereby modulating gene expression and metabolic pathways. In the context of biofilm formation, sRNAs employ diverse regulatory mechanisms, including up- or downregulation of gene expression, modulation of enzyme activity, and control of signal transduction pathways (30). For example, in *Escherichia coli*, the two-component sRNA system CsrB–CsrC regulates the activity of the global regulator CsrA; overexpression of CsrB/C suppresses CsrA function, thereby enhancing biofilm formation (31). Similarly, in *Pseudomonas aeruginosa*, the sRNA *pqsS* indirectly modulates biofilm architecture by regulating endogenous levels of the *Pseudomonas* quinolone signal PQS (32). In our study, crystal violet semi-quantitative assays, combined with scanning electron microscopy, were employed to evaluate the biofilm-forming capacities of *B. amyloliquefaciens* LPB-18 and its mutant strains LPB-18N and LPB-18P, revealing substantial differences in their biofilm synthesis abilities.

RNA–RNA interactions are central to metabolic regulation through gene expression control, with computational modeling and prediction serving as key methodologies in sRNA research (33). Modern high-precision prediction algorithms typically incorporate two critical parameters: hybridization energy, which reflects the stability of the intermolecular duplex, and binding probability, which estimates the likelihood of forming thermodynamically favorable secondary structures between interacting sequences (34). Among these tools, the IntaRNA 2.0.3 model has become a benchmark for predicting RNA interactions in both prokaryotic and eukaryotic systems, demonstrating outstanding performance in analyzing genomic and RNA sequencing data sets. Its robust capability to pinpoint specific seed interaction sites between sRNAs and their target mRNAs has earned widespread recognition in the research community. In this study, high-throughput sequencing was employed to analyze the transcriptome of *B. amyloliquefaciens* LPB-18N. Integrated analysis of RNA-Seq data using IntaRNA 2.0.3, combined with Pearson correlation coefficient calculations, led to the identification of 13 biofilm-associated sRNAs. Further bioinformatic screening highlighted one candidate, designated *fen36*, as a potential regulator of biofilm synthesis. By incorporating Pearson correlation analysis, we improved the predictive accuracy of IntaRNA 2.0.3, and this combined strategy substantially enhanced the reliability of our findings.

This study highlights the pivotal regulatory functions of non-coding RNAs in bacterial adhesion and biofilm biosynthesis. In *Escherichia coli*, the sRNAs r*prA* and *omrAB* inhibit the transcription of the guanylate cyclase-encoding gene *ydaM*, whose product, c-di-GMP, subsequently activates expression of the curli fimbriae master regulator *csgD* (35). By indirectly modulating *csgD*, these sRNAs influence both biofilm formation and dispersal. The CsrA–CsrB–CsrC regulatory system also plays a central role in governing biofilm biosynthesis in *E. coli* (36). In *Mycobacterium*, transcriptomic analyses have identified the non-coding RNA ncBCG427, which is positively correlated with biofilm formation and lipid metabolism (37). More recently, *Pseudomonas aeruginosa*, an opportunistic nosocomial pathogen, was shown to employ the novel sRNA AmiL to coordinate biofilm formation, swarming motility, and cytotoxic metabolism (38). To investigate the biological functions of *fen36*, we constructed scarless knockout (LPB-18NΔ*fen36*) and overexpression (LPB-18N::*fen36*) strains of *B. amyloliquefaciens*. Phenotypic characterization revealed that *fen36* acts as a positive regulator of biofilm synthesis: LPB-18N::*fen36* exhibited a 3.59-fold increase in biofilm production compared to the wild-type LPB-18N. Moreover, LPB-18N::*fen36* displayed enhanced motility on semisolid surfaces. *Bacillus* spp. coordinate biofilm formation and motility through an integrated regulatory network centered on Spo0A, the SinI–SinR module, and the DegS–DegU two-component system, which together create a bistable decision-making switch between the sessile and motile states. These regulators interact with post-transcriptional modulators, suggesting that *fen36* may function as an intermediate node linking sRNA-mediated control to the classical transcriptional hierarchy. Notably, overexpression of *fen36* did not affect fengycin biosynthesis, confirming its specific role in regulating biofilm formation while preserving secondary metabolite production.

**Fig 6:**
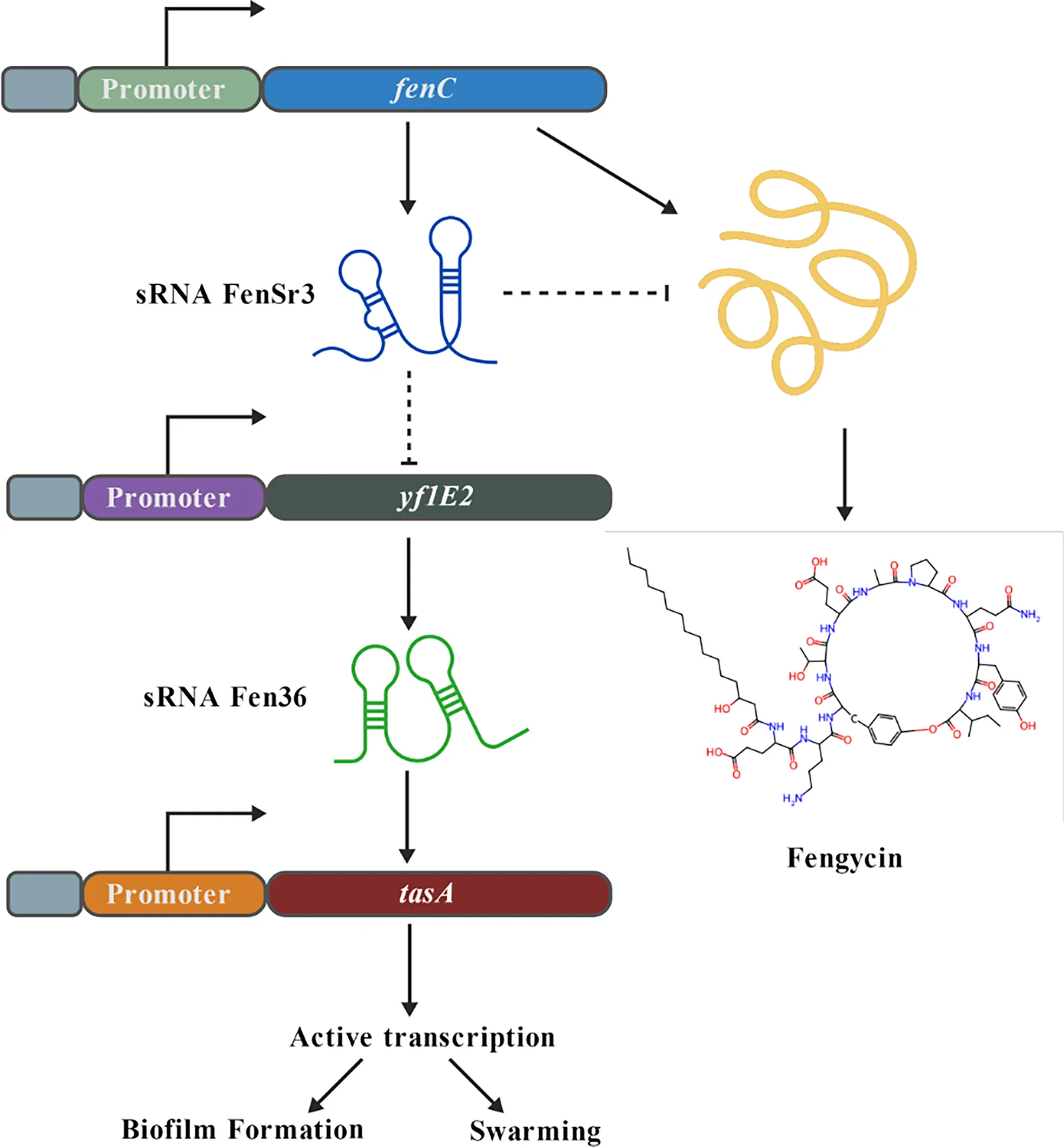
Schematic representation of the putative *fenSr3-fen36-tasA* regulatory cascade governing biofilm biosynthesis. Created with BioGDP.com (44). Solid arrows indicate activation or positive regulation, while blunt-ended lines represent repression. Dashed arrows denote predicted or indirect regulatory effects inferred from RNA–RNA interaction predictions (IntaRNA) or from prior literature. Components of the *tasA–sipW–tapA* operon are shown as structural genes responsible for amyloid fiber assembly. The proposed model integrates results from transcriptomic analysis, phenotypic assays, and bioinformatic predictions to describe the dual sRNA regulatory cascade (*fenSr3*→ *fen36*→ *tasA*) that controls multicellular behavior in *B. amyloliquefaciens*.

Further validation using a dual-plasmid reporter system demonstrated that sRNA *fen36* likely binds to the 5′ untranslated region (UTR) of the amyloid fiber protein-coding gene *tasA*, thereby enhancing its transcription. This finding is consistent with established paradigms of bacterial post-transcriptional regulation, in which 5′ UTRs frequently contain cis-regulatory elements that influence translation initiation and mRNA stability. Indeed, recent studies across diverse *Bacillus* species have shown that sRNAs often target the 5′ UTRs of structural genes to fine-tune biofilm matrix production under changing environmental conditions (39). Our data further revealed that the expression of sRNA *fen36* is negatively regulated by another regulatory sRNA, *fenSr3*. This hierarchical sRNA–sRNA interaction resembles previously described cascades in bacterial regulatory networks. For instance, in *Rhodobacter sphaeroides*, the stress-induced sRNA *srs*R directly base-pairs with *ups*M, an sRNA derived from the 5′ UTR of the *dcw* cell wall synthesis gene cluster. This complex recruits RNase E and induces conformational changes through synergistic sRNA–mRNA–RNase E interactions, thereby modulating cell proliferation and division (40). Temporal transcript profiling in our study demonstrated that *fenSr3* functions as a master repressor of both the biofilm synthesis operon (*tasA-sipW-tapA*) and *fen36*. Such repression suggested that *fenSr3* acts as a molecular gatekeeper, ensuring that biofilm initiation occurs only under appropriate physiological or environmental conditions. Similar regulatory mechanisms have been reported in *B. amyloliquefaciens*, where non-coding RNAs adjust exopolysaccharide and amyloid fiber production in response to nutrient availability (41). The “*fenSr3–fen36–tasA*” regulatory cascade uncovered in *B. amyloliquefaciens* LPB-18N represents a sophisticated, multi-tiered control system that optimizes biofilm biosynthesis. This model is supported by growing evidence that sRNA networks play central roles in coordinating biofilm development across bacterial species. For instance, in *Staphylococcus aureus*, RNAIII integrates multiple regulatory signals to control both virulence and biofilm-associated genes (42). Similarly, in pseudomonads, sRNAs such as RsmZ and RsmY participate in feedback loops that fine-tune biofilm formation in response to surface attachment and quorum-sensing signals (43). The proposed regulatory framework (Fig. 6) illustrated that *B. amyloliquefaciens* LPB-18N employs an integrated sRNA cascade to regulate biofilm development. This work added a new dimension to our understanding of the genetic basis of biofilm regulation in beneficial rhizobacteria and highlights promising targets for enhancing root colonization and persistence in agricultural applications through sRNA-based engineering.

In summary, this study conducted a comprehensive analysis of sRNA *fen36*-mediated regulation of biofilm synthesis through molecular target prediction, functional validation, and physiological characterization. It also provided new insights for the optimization of biofilm metabolic pathways and establishes a theoretical basis for further studies on sRNA-mediated post-transcriptional regulation in biofilm formation.

## MATERIALS AND METHODS

### Microorganisms and cultivation conditions

The experimental strains and plasmids used in this study were shown in Table 3. *B. amyloliquefaciens* LPB-18, *Escherichia coli* DH5α, and *Escherichia coli* BL21 were cultivated in Luria-Bertani (LB) medium at 33°C and 180 rpm. The strains LPB-18, LPB-18N, and LPB-18P were cultivated in LB medium (beef extract 5 g/L, peptone 10 g/L, yeast extract 5 g/L, NaCl 5 g/L, and glucose 10 g/L) at 33°C and 180 rpm for seed culture.

**TABLE 3:** All bacterial strains and plasmids used in this work

Strain or plasmids	Relative properties	Source
*B. amyloliquefaciens* LPB-18	Wild-type strain	Laboratory stock
LPB-18N	*fenSr3* deletion strain, a derivative of strain LPB-18	Laboratory stock
LPB-18P	LPB-18 harboring pHT-*fenSr3*	Laboratory stock
LPB-18N∆*fen36*	*fen36* deletion strain, a derivative of strain LPB-18N	Current study
LPB-18N::*fen36*	LPB-18N harboring pHT-*fen36*	Current study
*Escherichia coli* DH 5α	Competence	Laboratory stock
pCBS	pMAD with minor modification. The 3928-6049 bases in pMAD were removed, and Pamy, SamyE, and lacZ were added in that location. Ap^r^ Em^r^ (8102 bp)	Laboratory stock
pCBS-∆*fen36*	pCBS with *fenSr3* deletion box. Ap^r^ Em^r^	Current study
pHT43	*Escherichia coli* and *Bacillus subtilis* shuttle expression vector, Pgrac, Ap^r^ Cm^r^	Laboratory stock
pHT43-*fen36*	*fen36* expression vector	Current study
*Escherichia coli* BL 21	Competence	Laboratory stock
pET28-eGFP	Large intestine fluorescent plasmid; Kan	Laboratory stock
pET28-eGFP::*tasA*	pET28-eGFP with *tasA*; Kan	Current study
pET28-eGFP::*fenSr3*	pET28-eGFP with *fenSr3*; Kan	Current study

### Analysis of biofilm synthesis capacity of different strains

Two milliliters of LB liquid medium was added to sterile 96-well plates, along with 1 mL of seed solution from strains LPB-18, LPB-18N, LPB-18P, LPB-18N, LPB-18N∆*fen36,* and LPB-18N::*fen36*, each with an optical density at 600 nm (OD_600_) of approximately 2. The strains were inoculated using a pipette gun and subsequently enriched and cultured on a constant-temperature incubator shaker (CHANGZHOU HENGLONG YIQI, ZHP-100) at 33°C and 100 rpm for 3 h. Subsequently, the plates were transferred to a constant-temperature incubator (CHANGZHOU HENGLONG YIQI, DNP-9272) at 33°C for static culture for 48 h. All experiments were performed with six independent biological replicates (*n* = 6). Biofilm formation and motility assays were performed at 33°C to reflect the optimal physiological growth conditions of *B. amyloliquefaciens*.

### Transcriptome analysis screening for biofilm synthesis-associated sRNAs

Overnight cultures of the strains were adjusted to a consistent initial cell density (OD_600_ = 1.0) prior to inoculation. The same inoculum concentration was used for strains LPB-18 and LPB-18N to ensure that observed differences in biofilm formation were attributable to genetic variation rather than differences in the initial bacterial cell density. Overnight cultures of strain LPB-18 and strain LPB-18N were inoculated into fresh LB medium at a ratio of 1:100, cultured to the logarithmic phase, and the bacteria were collected. Total RNA was isolated by using the Bacteria Total RNA Isolation Kit (Sangon Biotech, B518625-0100). The quality of the total RNA was identified by RNA electrophoresis (LIUYI, DYY-2C), which should be basically free of degradation and genome contamination. The total RNA was subjected to high-throughput transcriptome sequencing using the Illumina HiSeq 2500/Miseq platform (Illumina, 2500).

### Bioinformatics analysis and independent transcription analysis

IntaRNA 2.0.3 was employed to predict sRNAs associated with biofilm synthesis and to analyze the binding ability of the sRNA *fenSr3* to genes encoding key components of the biofilm extracellular matrix. To ensure comparability with published *Bacillus* sRNA–mRNA interaction studies, the seed length threshold was set to ≥8 nucleotides and the maximum interaction free energy (ΔG) cutoff to ≤ −4.8 kcal/mol. These values have been commonly applied in previous reports to identify biologically meaningful RNA–RNA interactions while minimizing false positives (45, 46). The minimum accessibility energy was left at default settings, and predicted duplexes were visualized using RNAup and VARNA. The calculation method of IntaRNA 2.0.3 is as follows: ("--seedBP 8, --tAccW 140, --tAccL 70, --outMaxE −4.8, -m H"), based on the premise that the primary binding site of sRNAs is located in the 5′ UTR region of target genes. Therefore, the relationship pairs screened for sRNA binding at the translation initiation site of target genes, ranging from −39 to +19, were the final results. The predicted sRNA expression in different strains was verified by RT-qPCR.

### Functional validation of sRNA *fen36* from *B. amyloliquefaciens* LPB-18N

#### Construction of the sRNA *fen36* knockout mutant strain LPB-18NΔ*fen36*

To generate a markerless deletion of *fen36*, the upstream (355 bp) and downstream (557 bp) flanking sequences were amplified from the *B. amyloliquefaciens* LPB-18 genome and fused by overlap extension PCR to obtain a 930 bp fragment. The fusion product was inserted into the temperature-sensitive plasmid pCBS (digested with BamHI and EcoRI) using the ClonExpress II One Step Cloning Kit (Vazyme). The recombinant plasmid (pCBS-∆*fen36*) was first transformed into *E. coli* DH5α for propagation and verified by colony PCR and double digestion.

The confirmed plasmid was subsequently introduced into *B. amyloliquefaciens* LPB-18N by electroporation. Transformants were selected on LB agar plates containing erythromycin and cultured at 42°C to facilitate the first homologous crossover, producing single-crossover mutants. These mutants were then cultured at 30°C to promote the second crossover, resulting in successful markerless knockout strains.

#### Construction of the sRNA *fen36* overexpression strain LPB-18N::*fen36*

For overexpression of *fen36*, the full-length *fen36* sequence was amplified from the *B. amyloliquefaciens* LPB-18 genome. The PCR product was cloned into the shuttle vector pHT43 under the control of the Pgrac promoter using the ClonExpress II One Step Cloning Kit (Vazyme Biotech Co., Ltd., Nanjing, China). The recombinant plasmid (pHT43::*fen36*) was verified by colony PCR and restriction digestion following transformation into *E. coli* DH5α.

The confirmed plasmid was then introduced into *B. amyloliquefaciens* LPB-18N via electroporation. Transformants were selected on LB agar plates supplemented with chloramphenicol and verified by PCR. Positive colonies were inoculated into LB medium, and *fen36* overexpression was induced with 1 mM IPTG, generating the strain LPB-18N::*fen36*.

### Phenotypic assays of different engineered strains

#### Determination of growth curves

The strains of LPB-18N, LPB-18NΔ*fen36*, and LPB-18N::*fen36* were individually inoculated into 100 mL of seed medium and cultured overnight at 33°C with shaking at 180 rpm. Subsequently, each culture was transferred into fresh LB medium at a 1% (vol/vol) inoculum size and incubated under the same conditions (33°C, 180 rpm). Samples were collected every 3 h to measure the optical density at 600 nm (OD₆₀₀). All experiments were performed in triplicate. Growth curves were plotted with time on the x-axis and OD₆₀₀ values on the y-axis.

#### Biofilm phenotyping

The strains of LPB-18, LPB-18N, LPB-18N∆*fen36*, and LPB-18N::*fen36* were inoculated into 96-well plates containing tryptic soy broth (TSB; tryptone, 17.0 g/L; soytone, 3.0 g/L; K₂HPO₄, 2.5 g/L; NaCl, 5.0 g/L; glucose, 2.5 g/L). The plates were subsequently incubated statically at 33°C, and biofilm formation was assessed after 48 h. The quantity of biofilm produced was semi-quantified using a crystalline violet staining method. Biofilm formation ability of different strains was semi-quantitatively measured every 6 h during the culture process using the crystal violet staining method (each strain was repeated in 20 wells). First, the culture medium was carefully aspirated from the deep-well plate using a syringe, ensuring the integrity of the upper biofilm was maintained. The wells were then washed three times with sterile PBS buffer and air-dried at room temperature. Next, 1 mL of formaldehyde was used to fix the biofilm (immersing the biofilm completely), and the plate was left to stand for 5 min. The formaldehyde was then aspirated, and this process was repeated 1–2 times, after which the plate was dried in a 40°C oven. Finally, the biofilm was stained with 1 mL of 0.5% crystal violet solution for 10 min. After staining, the wells were washed with absolute ethanol, and the ethanol wash was collected. The absorbance at 590 nm was measured to evaluate the biofilm formation ability of different strains, with absolute ethanol as the blank control. Each sample was measured in triplicate. The biofilm formation ability of the different strains was then assessed based on the absorbance values.

#### Observation of biofilm microstructure

Aliquots (10 mL) of 36 h stationary culture broths of LPB-18, LPB-18N, and LPB-18N△*fen36* were centrifuged at 2,500 × *g* and 4 °C using a pre-cooled centrifuge (Beckman Coulter, Allegra X-30R). The supernatant was discarded, and the pellets were resuspended and fixed with 5 mL of 2.5% glutaraldehyde solution for 4 h. The bacterial cells were then washed three times with 1× PBS buffer. Subsequently, the samples were dehydrated through a graded series of ethanol solutions (30%, 50%, 70%, 80%, 90%, and 95%). Each step lasted 10 min, followed by two additional 10-min treatments with absolute ethanol. Finally, the samples were dropped onto glass slides and air-dried in a laminar flow hood. The biofilm microstructure of the samples was observed by scanning electron microscopy (HITACHI, S-3000N), which was partially entrusted to the Analysis and Testing Center, Huaiyin Institute of Technology.

#### Swarming motility assay

The solid LB agar medium should be configured to a concentration of 0.5%. Then, 5 mL of the activated cultures of *B. amyloliquefaciens* LPB-18N, *B. amyloliquefaciens* LPB-18N∆*fen36*, and *B. amyloliquefaciens* LPB-18N::*fen36* should be aspirated into the center of the plate. The plate should then be placed in a constant-temperature incubator set to 33°C for static incubation. Cell motility should be recorded 24 h later.

#### Determination of bacteriostatic activity

The inhibitory effects of wild strain LPB-18, strain LPB-18N, strain LPB-18N∆*fen36*, and strain LPB-18N::*fen36* against *Fusarium spinosum* were evaluated using a plate standoff assay, which was performed as follows: holes were punched using a sterilized 6 mm perforator in activated *Fusarium spinosum* plates, and the plugs were inoculated at the center of the PDA plates. A pipette gun was utilized to dispense 5 μL of bacterial solution onto both sides of the pathogenic fungal plug, with *B. amyloliquefaciens* LPB-18 serving as the control. Following the drying of the bacterial solution, it was sealed with sealing film and placed in dark culture at 28°C for a period of 7 days. The experiment was repeated on three separate occasions, and the diameter of the ring of inhibition was measured.

#### Determination of yield of mutant strain fengycin

The determination of fengycin was conducted following a previous methodology (47). Briefly, the engineering strains were introduced into the fermentation medium at an inoculum concentration of 3% and incubated at a constant temperature of 33°C for a period of 48 h. Subsequently, the cell-free supernatants (200 mL) were obtained by centrifugation at 6,500 × *g* for 15 min at 4°C. After adjusting to pH 2.0 with 6 M hydrochloric acid, the supernatant was kept overnight at 4°C and then centrifuged at 6,500 × *g* for 15 min at 4°C. The precipitate was collected and dissolved in 10 mL methanol. Lipopeptide extracts were filtered through a 0.22 μm bacterial filter for HPLC examination (Shimadzu, Japan). The mobile phase A was deionized water containing 0.1% trifluoroacetic acid, and mobile phase B was acetonitrile containing 0.1% trifluoroacetic acid. The flow rate was maintained to be 0.6 mL/min under the following conditions: 0–15 min, 70% A to 55% A, 30% B to 45% B; 15–55 min, 55% A to 45% A, 45% B to 55% B. Monitoring was conducted at 205 nm using a UV detector.

### Validation of the *in vivo* target of sRNA *fen36*

#### Construction of pET28-eGFP::*tasA* and pET28-eGFP::*fenSr3* green fluorescent protein expression vectors

Using the *Bacillus amyloliquefaciens* Y2 genome as a reference, primers targeting the upstream regions of the *fenSr3* and *tasA* promoters (incorporating homologous arms for the vector) were designed using Primer 5.0 and commercially synthesized (Sangon Biotech, Shanghai). Genomic DNA was extracted from the wild-type strain LPB-18 using a Bacterial DNA Kit (OMEGA, D3350, USA) and served as the template for PCR amplification to obtain the target promoter fragments. Plasmid pET28-eGFP was extracted, its quality was verified by agarose gel electrophoresis, and it was linearized via double digestion with NdeI and XbaI. The promoter fragments and linearized vector were then assembled using a seamless cloning kit (BBI, 2 × Ezmax Universal CloneMix). The assembled products were transformed into *E. coli* DH5α competent cells for positive clone selection. Positive clones were enriched in culture and verified for successful plasmid construction by double digestion analysis.

#### Construction of a dual-plasmid system

The successfully verified fusion expression vectors, pET28-eGFP::*tasA* and pET28-eGFP::*fenSr3*, were individually transformed into *E. coli* BL21 competent cells. Subsequently, the overexpression vector pHT43-Fen36 was introduced into each of these strains. This generated dual-plasmid systems harboring both pET28-eGFP::*tasA*/pHT43::*fen36* and pET28-eGFP::*fenSr3*/pHT43::Fen36. Following antibiotic selection, transformants were induced with IPTG and cultured in a shaker at 37°C for 24 h. The fluorescence intensity of the fermentation broth was then measured using a multi-mode microplate reader with excitation at 488 nm and emission at 540 nm. Each experiment was performed in triplicate, using cells containing pET28-eGFP as a blank control. Dual-plasmid reporter assays were conducted in *Escherichia coli* at 37°C to ensure efficient expression of the heterologous plasmid-based system.

#### Analysis of sRNA-mediated regulatory role in biofilm formation

To further analyze the regulatory relationships between sRNA *fenSr3*, sRNA *fen36*, and the biofilm-associated *tap* operon (*tasA-sipW-tapA*), the expression levels of *fen36*, *fenSr3*, and the *tasA-sipW-tapA* gene cluster were quantified in different strains: LPB-18, LPB-18N, and LPB-18N::*fen36*. Real-time expression profiles were constructed for each gene across these strains to investigate the interactions between the sRNAs and their regulation of target genes. All strains tested were cultured in TSB medium at 33°C for 24 h. Total RNA was then extracted from each culture and used for gene expression analysis. The specific primers designed for the RT-qPCR amplification are listed in Table 4. RT-qPCR analyses were performed using 16S rRNA as the internal reference gene, and all measurements represent the mean ± SD from three independent biological replicates (*n* = 3).

**TABLE 4:** Primer sequences used for RT-qPCR analysis in this study

Gene	Primer	Sequence (5′–3′)
*fen36*	*fen36*-F	TTCACATTCTGCACGGCAT
*fen36*	*fen36*-R	AATCATTCAGAATCGTTTTGGCT
*fenSr3*	*fenSr3*-F	TTCGGCAGTTGCTTAGACAGTGAAG
*fenSr3*	*fenSr3*-R	GAGCCTGGACGGTGTGAAAGAAG
*tasA*	*tasA*-F	GGATAAAGAAGACACGGAC
*tasA*	*tasA*-R	GAATCCATGCCGCTCTT
*sipW*	*sipW*-F	ATGATGCAGGCAAAGA
*sipW*	*sipW*-R	CGAAATGTACAAAGCG
*tapA*	*tapA*-F	TCCGTAACGGAGACTTC
*tapA*	*tapA*-R	GCCGTTCTATACGAAGTTC
16S rRNA	16S-F	ACGGTCGCAAGACTGAAACT
16S rRNA	16S-R	TAAGGTTCTTCGCGTTGCTT
